# Variations in Morphological Characters and Antioxidant Potential of Different Plant Parts of Four *Ziziphus* Mill. Species from the Cholistan

**DOI:** 10.3390/plants10122734

**Published:** 2021-12-12

**Authors:** Muhammad Umair Riaz, Muhammad Ali Raza, Amjad Saeed, Mukhtar Ahmed, Tanveer Hussain

**Affiliations:** 1 Department of Forestry, Range and Wildlife Management, Faculty of Agriculture and Environment, The Islamia University of Bahawalpur, Bahawalpur 63100, Pakistan; cumair84@yahoo.com (M.U.R.); amjadsaeed101@gmail.com (A.S.); 2 National Research Center of Intercropping, The Islamia University of Bahawalpur, Bahawalpur 63100, Pakistan; razaali0784@yahoo.com; 3 Department of Agronomy, PMAS-Arid Agriculture University, Rawalpindi 46300, Pakistan

**Keywords:** bioactive, desert, irrigated, flavonoid, phenol, phytochemistry

## Abstract

Genus *Ziziphus* (*Z.*) contains various important species in tropical and subtropical regions that are globally famous for their food and medicinal uses. However, no comprehensive study was available on the morphology and phytochemistry of *Ziziphus* species, mainly under different growth conditions, i.e., irrigated and desert (Cholistan). Therefore, this study was carried out to evaluate the morphological and phytochemical characteristics of *Ziziphus* species, i.e., *Z. jujuba*, *Z. mauritiana*, *Z. spina-christi*, and *Z. nummularia,* found in the irrigated and desert conditions. Our results revealed significant variations for most of the measured parameters, showing a large-scale diversity among *Ziziphus* species under irrigated and desert conditions. Specifically, *Ziziphus* species showed better morphology of all measured parameters of leaves and fruits under irrigated conditions compared to desert conditions, indicating that the optimum water availability in irrigated conditions improved the morphological parameters of *Z*. species. Meanwhile, among all *Ziziphus* species, the maximum leaf length (7.4 cm), leaf width (4.1 cm), leaf area (30.6 cm^2^), and leaf petiole length (1.3 cm) were observed for *Z. jujuba*, and the highest leaf dry weight (55.4%) was recorded for *Z. mauritiana*. Similarly, the highest fruit length (3.9 cm), fruit stalk length (1.5 cm), fruit diameter (3.6 cm), fruit width (3.8 cm), fruit area (66.1 cm^2^), seed length (2 cm), and seed diameter (1.1 cm) were measured for species *Z. jujuba*, while the maximum fruit dry weight (49.9%) and seed width (1.4 cm) were recorded for species *Z. nummularia*. Interestingly, compared to irrigated conditions, higher values of bioactive contents, i.e., phenol, flavonoid, and antioxidant activity, in fruits and leaves of *Ziziphus* species under desert conditions indicated the positive impact of desert climate on the phytochemistry of the *Z.* plants. Among *Ziziphus* species, *Z. nummularia* accumulated the maximum fruit phenols (304.4 mg GAE/100 g), leaf phenols (314.2 mg GAE/100 g), fruit flavonoids (123.7 mg QE/100 g), and leaf flavonoids (113.4 mg QE/100 g). Overall, this study demonstrated the significant morphological and phytochemical variations of the *Ziziphus* species under irrigated and desert conditions, which could be utilized for future studies to improve the production and medicinal potential of the *Ziziphus*, especially in desert areas.

## 1. Introduction

Genus *Ziziphus* Mill., commonly called Ber, comprises deciduous or evergreen trees and shrubs, widely distributed in the tropical and sub-tropical areas of the world [1]. *Ziziphus* belongs to the family *Rhamnaceae*, which contains 100 species in the Old-world (i.e., African, European, and Asian) and New-world (i.e., America). *Ziziphus* species are pantropical and paraphyletic and generally split into three different geographical lineages [2]. Recent studies revealed that these lineages are categorized into genus *Condaliopsis* and *Sarcomphalus*, regarded as new world *Ziziphus* and genus *Ziziphus*, *considered* old-world *Ziziphus* [3,4]. *Ziziphus* species are reported as a good food source, especially for those who live in desert areas, because it fulfills nutritional needs and maintains health and food security and economic welfare [5]. In addition, these species contain a high content of several minerals (i.e., vitamin C, phosphorus, calcium, and iron), amino acids (i.e., asparagine, glutamic acid, arginine, aspartic acid, serine, glycine, and threonine), and sugars (i.e., sucrose, fructose, glucose, and starch) [6]. Furthermore, local communities eat *Ziziphus* fruit (Ber) directly in fresh or dry form. Numerous companies use its fruit to make butter, pastes, and flour [7]. Its leaves are the source of vegetables and medicine for treating various diseases (i.e., diabetes, asthma, and depression). It is also a great source of fodder for livestock due to its high nutritional value [1]. Notably, *Ziziphus* species are drought-tolerant plants usually found as natural vegetation in the deserts [8] and exist in pastures, coastal, and wet mountain regions [9]. These species comprise diverse plants from shrubs to small or medium-sized trees, having an extensive range of canopy morphology such as spreading, semi-erect, and erect, and its height ranges from 4 to 16 m [10]. Z. leaves, fruits, and flowers have great diversity in shape, color, and taste [11,12,13].

Environmental factors, i.e., temperature, light, and rainfall, affect plant growth and development in several ways [14,15]. However, trees can cope with changing environmental conditions by alternating their organs and tissues [16]. In this perspective, due to *Ziziphus* species’ drought-tolerant and heat resistant characteristics [17], they can grow well under stress conditions, i.e., high temperature and low rainfall, thus, are responsible for diverse morphological patterns to mitigate these effects [18]. Such interspecific patterns between plant traits and climatic factors were previously reported by many scientists [19,20,21], which strongly correlate mean annual rainfall, nutrient availability, and light intensity with leaf shape, leaf area, and leaf size. However, there is no study available in the current literature on determining the morphological characteristics of *Ziziphus* plants under different growing conditions, especially in Cholistan (high-temperature region, where total rainfall is less than total evapotranspiration). Morphological characteristics are the most vital determining indices for better characterization, taxonomic classification, and agronomic value of the plants while examining the genetic diversity. Notably, the information about the phenotypic diversity of *Ziziphus*, particularly the native desert species (e.g., *Ziziphus jujuba*, *Ziziphus mauritiana*, *Ziziphus spina-christi*, and *Ziziphus nummularia*) is minimal. Therefore, a comprehensive study is needed to determine the morphological plasticity of each component of the *Ziziphus* species present in the desert. Such information is also crucial for the commercial production of Beri with better yield, lower inputs, and improved nutrition [2,22]. In addition, this type of study could also enhance our understanding of adaptations and responses of morphological traits in different desert environments, which could help us better utilize the desert lands under changing climatic conditions.

*Ziziphus* species are mainly used as traditional medicine and possess important phytochemicals, i.e., flavonoids, phenols, triterpenoids, and alkaloids [23,24,25,26], which play a promising role in the treatments of many diseases, i.e., cancer, ulcer, and inflammation [27,28], due to their therapeutic prospective, i.e., antioxidant properties [29,30]. Previously, scientists reported that the antioxidant compound in *Ziziphus* species can inhibit or delay the oxidative damage caused by reactive oxygen species in humans and animals, thus reducing several ailments, i.e., inflammation and aging [31,32,33]. Similarly, flavonoids and phenols are natural antioxidants, which possess a broad range of multiple biological activities and unique structures, playing important roles in minimizing the risks of oxidative stress-based complaints and infectious ailments [34,35,36]. These findings conclude that *Ziziphus* species are a rich source of phytochemicals for humans and animals. However, how desert conditions affect the quantity of phytochemicals in *Ziziphus* species is rarely investigated. Therefore, we hypothesized that desert conditions could affect the phytochemistry of *Ziziphus* species, ultimately influencing the therapeutic prospective of these species. A morphological characterization is an important tool in identifying species and determining phenotypic diversity among various plant species. The present research was designed to investigate the variations of (i) morphological characters (i.e., qualitative and quantitative), (ii) bioactive contents (i.e., phenols, flavonoids), and antioxidant potential of different parts of *Ziziphus* species (*Z. jujuba*, *Z. mauritiana*, *Z. spina-christi*, and *Z. nummularia*) under different growth conditions, i.e., irrigated and desert (Cholistan). In addition, these types of phytochemical investigation of *Ziziphus* species can help to quantify the different bioactive compounds and their potential use as medicine and will reduce the pressure on other medicinal plant species, which are being used in traditional medicines, thus, providing an alternative against different ailments.

## 2. Materials and Methods

### 2.1. Characteristics of the Study Area

**Figure 1:**
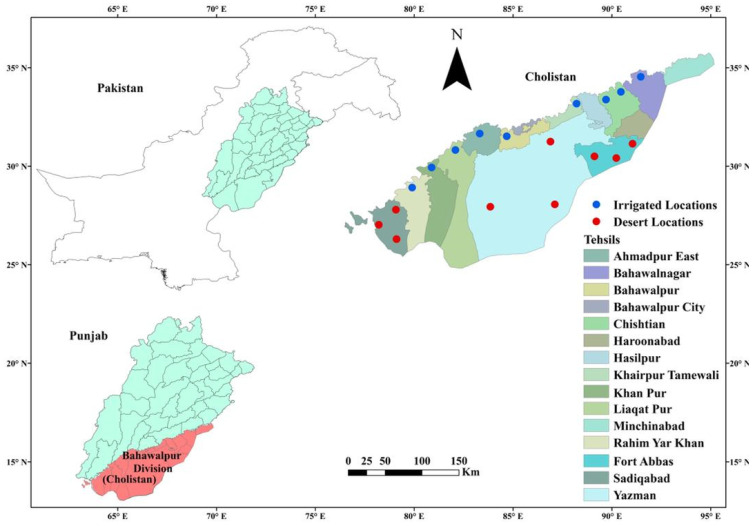
Study area map.

This study was conducted for one year in the Cholistan desert with a hot and arid climate, extended over a vast area of 2600 km^2^, adjacent to the district Bahawalpur, Southern Punjab, Pakistan (Figure 1). Cholistan desert lies between 75°24′ E and 27°42′ N [37]. The average rainfall of this region ranges from 100 to 250 mm, falling mainly during the monsoon season. Mean summer and winter temperature ranges from 34 to 38 °C and 14 to 16 °C, respectively. During summer (May–July), the maximum temperature reaches 52 °C while the minimum temperature during the winter falls below zero (Metrology Department, Government of Punjab, Pakistan). The soil of the Cholistan desert is a mixture of sandy silt and sandy clay [38].

### 2.2. Morphological Characterization

Seventy-two samples (24 plants; 12 plants from the desert and 12 plants from the irrigated area) of fruits, leaves, and seeds of four *Ziziphus* species were collected randomly from three districts, i.e., Bahawalnagar, Bahawalpur, and Rahim Yar Khan, of the Cholistan desert and Irrigated plains, providing the opportunity to study the relationship between environmental factors and morphological traits of different *Ziziphus* species under the desert and irrigated conditions. Plants with similar morphological characters were selected for the measurement of morphological parameters. Fruit and seed collection was done at the final stage of fruit ripening (March) from the naturally grown old plants, while leaves were collected before the fruit set (October–November). At each location in the irrigated and desert conditions, three individual plants of each *Ziziphus* species (*Z. jujuba*, *Z. mauritiana*, *Z. spina-christi*, and *Z. nummularia*) were selected to study the 27 morphological characters [2]. Fruit, leaves, and seed were collected to measure the quantitative and qualitative characteristics of *Ziziphus* species. For qualitative measurements, selected plant parts of each species were subjected to visual analysis to identify traits according to the available key for each parameter. Fruit shape, fruit apex, fruit base, fruit color, stalk color, leaf color, leaf shape, leaf veins, leaf base, leaf apex, leaf margin, leaf petiole color, and leaf petiole surface were recorded to determine the qualitative morphological characters of *Ziziphus* species. Furthermore, for the measurement of quantitative traits, three random fresh samples of fruits and three random fresh samples of leaves of each species from each growing condition were sampled [18]. To calculate fruit and leaf dry weight, 100 g of fresh fruit samples and leaves were used. Fruit weight (g), fruit length (cm), fruit width (cm), fruit diameter (cm), fruit area (cm^2^), stalk length (cm), leaf length (cm), leaf width (cm), leaf area (cm^2^), leaf weight (g), and petiole length were recorded to evaluate the quantitative characters of *Ziziphus* species [8]. Fruit, leaves, and seed sizes were measured using a vernier caliper, while the electrical weigh-balance was used to measure the weights of fruits and leaves [39]. Furthermore, leaf area was measured by multiplying leaf length with leaf width. The following equation determined fruit area:

Fruit Area = 2πr^2^ + 2πrl

where, π = 3.14, r = fruit radius, and l = fruit length.

### 2.3. Preparation of Fruits and Leaves for Chemical Analysis

Fresh fruit and leaf samples of four *Ziziphus* (*Z*) species (*Z. jujuba*, *Z. mauritiana*, *Z. spina*-*christi*, and *Z. nummularia*) were collected from the different growing conditions, i.e., irrigated and desert (Cholistan). Collected samples were analyzed for the determination of phytochemicals and antioxidant potential. Firstly, the collected samples were thoroughly washed with clean water to remove any contamination and then dried under the shade for about 15 to 17 days. After that, the leaf and fruit samples were ground and stored in zipped plastic bags for analysis in the laboratory. Subsequently, 200 g of plant material powder was mixed with 1000 mL of *N*-hexane (solvent) and kept soaked for two weeks at room temperature. During this duration, the soaked solution was shaken regularly every three days. A muslin cloth was used to filter out any coarse material from soaked material, and the obtained filtrate was boiled to evaporate the menstruum. Finally, a Whatman filter paper was used to filter the mixture, and the final extract was collected in a petri dish. A similar extraction method was repeated with methanol in place of *N*-hexane as a solvent [40]. *N*-hexane extract was used to measure the total phenolic and flavonoid contents, while methanolic extract was used to evaluate free radical scavenger DPPH (1, 1-diphenyl-2-picrylhydrazyl) activities.

### 2.4. Total Phenolic Content

The Folin-Ciocalteu reagent method was used for the determination of phenol quantity in plant extract (sample). *N*-hexane (0.2 mL) extract was mixed with the Folin reagent (750 μL) and 6% sodium carbonate Na_2_CO_3_ (750 μL), followed by a dark incubation of the mixture for 90 min. After dark treatment, the absorbance of the sample was calculated at 765 nm through a spectrophotometer. Gallic acid was used for the preparation of the standard curve (0.250 mg/L). Total phenolic content was measured from the standard curve and stated as milligrams (mg) of gallic acid equivalents (GAE) per 100 g of extract (mgGAE/100 g) [41].

### 2.5. Total Flavonoid Content

For the determination of flavonoids, the aluminum chloride colorimetric method was used; 1 mL of 2% aluminum chloride solution (AlCl_3_, 6H_2_O) was mixed with 1 mL *N*-hexane plant extract (sample). This mixture was incubated for 10 min at room temperature, and the absorbance of the sample was calculated at 420 nm through a spectrophotometer. Quercetin was used as standard (0–100 mg/L). Quantity of flavonoid was measured from the standard curve and stated as milligram (mg) of quercetin equivalents (QE) per 100 g of extract (mg QE/100 g) [42].

### 2.6. Free Radical Scavenger DPPH (1,1-Diphenyl-2-picrylhydrazyl) Activity

DPPH radical scavenging assay was implemented to measure the antioxidant potential of the plant extract [41]. A solution containing 0.004% 1,1-diphenyl-2-picrylhydrazyl (DPPH), 1 mL methanol, and 3 mL of plant extract was prepared and kept in the dark for 30 min. Then, the absorbance of the mixture was calculated at 517 nm with a spectrophotometer, and an extract-free solution was used as a control. A low absorbance of the reaction mixture highlighted a vast radical scavenging activity. For measuring the percentage inhibition of DPPH radical of the samples, the calculation was done through the following equation:

DPPH Inhibition (%) = {(Ab−As)/Ab} × 100

where Ab is the absorbance of blank, and As is the absorbance of the sample.

### 2.7. Statistical Analysis

Data were analyzed using computer software Statistix 8.1. Growing conditions were allocated to main plots while *Ziziphus* species were maintained in the sub-plots. Significant differences were determined using ANOVA in combination with the LSD (least significance difference) test to determine the differences among the plant species. The significance of differences was evaluated at *p* < 0.05 levels. Tables report the means of calculated means based on the three replicates per treatment.

## 3. Results

### 3.1. Qualitative Morphological Characters

#### 3.1.1. Fruit Morphological Parameters of *Ziziphus* Species under Irrigated and Desert Conditions

The morphological characterization of *Ziziphus* species in the present study revealed significant changes for various qualitative morphological traits among *Ziziphus* species under different growing conditions, i.e., irrigated and desert (Cholistan). Overall, the fruit shape was noticed as oval, orbicular, rounded, and ovate, and the fruit stalk color was observed as light green and dark green in all the studied species under both conditions. The fruit apex was found as retuse and truncate in *Z*. *jujuba*, oblate and rounded in *Z*. *mauritiana*, obtuse in *Z*. *spina*-*christi*, and oblate in *Z. nummularia* in both growing conditions. The fruit base was observed as rounded in *Z. jujuba* and *Z. spina-christi*, while in *Z. mauritiana* and *Z. nummularia*, it showed a flat pattern in desert conditions. In irrigated lands, the fruit base was shown as rounded and oval in *Z. jujuba*, orbicular in *Z. mauritiana*, rounded in *Z. spina*-*christi*, and flattened in *Z. nummularia*. Moreover, the color of the ripened fruit was yellowish-green to red in *Z. jujuba*, dark green in *Z. mauritiana*, light-green to yellow in *Z. spina*-*christi*, and yellow-green to red in *Z. nummularia* under both growing conditions (Table 1).

#### 3.1.2. Leaf Morphological Parameters of *Ziziphus* Species under Irrigated and Desert Conditions

Variations in the leaf morphological characters were recorded for the studied *Ziziphus* species in different growth conditions. Leaf shape was observed as oval and oblong in *Z. jujuba*, oval and lanceolate in *Z. mauritiana*, oval, ovate, and cordate in *Z. spina-christi* and, oval and rounded in *Z. nummularia* in desert conditions. In contrast, leaf shape was found as oval and oblong in *Z. jujuba*, oval, ovate, and cordate in *Z. mauritiana*, oval, ovate, and cordate in *Z. spina-christi* and, rounded and ovate in *Z. nummularia* in irrigated conditions. Leaf apex was an obtuse shape for all the studied species, except for the acute leaf apex in the *Z. spina*-*christi* in both growing conditions. In the desert conditions, the shape of the leaf base was measured as round and acute in *Z. jujuba* and *Z*. *mauritiana*, rounded and cordate in *Z. spina*-*christi*, and round only in *Z. nummularia*. Moreover, the shape of the leaf base was determined as cuneate and rounded in *Z. jujuba*, acute and rounded in *Z. mauritiana*, rounded and cordate in *Z. spina*-*christi*, and round in *Z. nummularia* in irrigated plains. Leaf margins showed no variation as all the species exhibited an entire shape in both growing conditions. However, leaf color varied among the studied species as *Z. jujuba* showed shining dark green leaves, *Z. mauritiana* showed light green leaves, and both *Z. spina*-*christi* and *Z. nummularia* showed dull green leaves in both growth conditions. Moreover, variations were shown in the surface of the leaf petiole, even sometimes in the same species of *Ziziphus*. Leaf petiole surface was detected as glabrous in *Z. nummularia* and pubescent in *Z. jujuba*. At the same time, some species (*Z. mauritiana* and *Z. spina*-*christi*) were covered with hairs in both growing conditions (Table 2).

### 3.2. Quantitative Morphological Characters

#### 3.2.1. Fruit Morphological Parameters of *Ziziphus* Species under Irrigated and Desert Conditions

In the current study, different growing conditions significantly (*p* < 0.05) changed the fruit length, fruit width, fruit diameter, fruit area, dry fruit weight, seed length, seed diameter, and seed width, while both growing conditions showed non-significant differences for fruit stalk length of *Ziziphus* species; data are shown in Table 3. The maximum fruit length (2.7 cm), fruit width (2.8 cm), fruit diameter (2.8 cm), fruit area (38.1 cm^2^), seed length (1.6 cm), seed diameter (1.0 cm), and seed width (1.4 cm) were noticed under irrigated conditions. In contrast, minimum fruit length (2.5 cm), fruit area (36.1 cm^2^), fruit width (2.6 cm), fruit diameter (2.6 cm), seed length (1.4 cm), seed diameter (0.9 cm), and seed width (1.2 cm) were observed in desert conditions. In addition, the highest (32%) and lowest (27%) values of dry fruit weight were measured in the irrigated and desert conditions, respectively.

Among *Ziziphus* species, the highest fruit length (3.9 cm), fruit stalk length (1.5 cm), fruit diameter (3.7 cm), fruit width (3.8 cm), fruit area (66.2 cm^2^), seed length (2.1 cm), and seed diameter (1.2 cm) were measured for species *Z. jujuba*. In comparison, the maximum dry fruit weight (50%) and seed width (1.3 cm) were recorded for species *Z. nummularia*, respectively. Whereas the minimum fruit length (1.6 cm), fruit width (2.1 cm), fruit area (17.9 cm^2^), fruit diameter (2.1 cm), seed length (1.1 cm), and seed diameter (0.8 cm) were noted for species *Z. nummularia*; while the lowest fruit stalk length (1.0 cm), and dry fruit weight (15%) and seed width (1.3 cm) were determined for *Z. mauritiana* and *Z. jujuba*, respectively, indicating that the different growing conditions caused a significant change in the morphology of *Ziziphus* fruit. Furthermore, the interactive results for *Ziziphus* species and growing conditions were significant for fruit length and seed diameter, while a non-significant interaction was found for fruit diameter, fruit width, fruit area, dry fruit weight, fruit stalk length, seed length, and seed width (Table 3).

#### 3.2.2. Leaf Morphological Parameters of *Ziziphus* Species under Irrigated and Desert Conditions

Growing conditions and Ziziphus species exhibited significant (*p* < 0.05) variations for leaf area, leaf length, leaf petiole length, leaf width, and leaf weight, as presented in Table 4. The highest leaf area (30.6 cm^2^), leaf length (7.4 cm), and leaf width (4.1 cm) were determined for *Z. jujuba*; while the maximum leaf petiole length (1.3 cm) and leaf dry weight (55%) were obtained for *Z. mauritiana* under irrigated conditions, suggesting that optimum water conditions can improve the tree physiology (net photosynthetic rate, nutrient, and water uptake from soil) of *Ziziphus* species by improving the leaf morphology, as observed in this study. Whereas the lowest leaf area (5.5 cm^2^), leaf length (2.6 cm), leaf petiole length (0.5 cm) and leaf width (2.1 cm), and leaf dry weight (38%) were noticed for *Z. nummularia* and *Z. spina-christi*, respectively, in desert conditions. Moreover, the interactive results for *Ziziphus* species and growing conditions were found significant for leaf dry weight, while it was found non-significant for leaf area, leaf length, leaf petiole length, and leaf width (Table 4).

### 3.3. Total Phenol and Flavonoid Contents in Fruits and Leaves of Ziziphus Species under Irrigated and Desert Conditions

In the current study, different growing conditions significantly (*p* < 0.05) altered the phytochemical (i.e., total phenol and flavonoid) contents in fruits and leaves of *Ziziphus* species; data are shown in Table 5. Compared with irrigated conditions, the relative increase in the total phenol and flavonoid contents in leaves and fruits of *Ziziphus* species were 3.6% and 3.9%, respectively, under desert conditions, suggesting that drought conditions favor the production and accumulation of phenol and flavonoid *Ziziphus* species.

In addition, among *Ziziphus* species, the highest fruit phenols (304.4 mg GAE/100 g), leaf phenols (314.2 mg GAE/100 g), fruit flavonoids (123.7 mg QE/100 g), and leaf flavonoids (113.4 mg QE/100 g) were measured for species *Z. nummularia*. Meanwhile, the lowest leaf phenols (234.5 mg GAE/100 g) and fruit phenols (207.6 mg GAE/100 g) were determined for species *Z. mauritiana*, while the minimum leaf flavonoids (87.9 mg QE/100 g) and fruit flavonoids (95.6 mg QE/100 g) were noticed for species *Z. spina-christi*. Furthermore, the interactive results for Ziziphus species and growing conditions were found significant for leaf flavonoids and fruit flavonoids, whereas it was found non-significant for leaf phenols and fruit phenols.

### 3.4. DPPH Scavenger Activities for the Fruits and Leaves of Ziziphus Species under Irrigated and Desert Conditions

In the current study, different climatic conditions significantly (*p* < 0.05) changed the antioxidant potential of leaves and fruits of *Ziziphus* species; data are presented in Table 5. The maximum % inhibition of DPPH in leaves (69%) and fruits (65%) were calculated under desert conditions. In contrast, minimum % inhibition of DPPH in leaves (58%) and fruits (53%) were measured in irrigated conditions.

Among Ziziphus species, the maximum % inhibition of DPPH in fruits (67%) and leaves (71%) were measured for species *Z. spina-christi*. Whereas the minimum % inhibition of DPPH in fruits (53%) and leaves (57%) were determined for species *Z. nummularia*, indicating that the different levels of water availability directly altered the antioxidant activity potential of *Ziziphus* plants. Furthermore, the interactive results for *Ziziphus* species and climatic conditions were significant and non-significant for % inhibition of DPPH in leaves and fruits, respectively.

## 4. Discussions

This study reveals that improved water conditions under irrigated areas, influenced by irrigation, have significant impacts on various morphological parameters of *Ziziphus* species. Compared to desert *Ziziphus* plants, the better morphology of all studied *Ziziphus* species in irrigated conditions resulted in large leaves and fruits of *Ziziphus* species. Similarly, variations in the fruit size of different *Ziziphus* species (*Z. jujuba* and *Z. spina-christi*) were also reported by Zeinelabdin [43] under different types of growing conditions in Sudan. However, the observed differences among the different *Ziziphus* species with respect to petiole surface were not matched with the previous findings reported by Almalki and Alzahrani [44]; these ontogenic changes in petiole surface were associated with genetic characters of *Ziziphus* species [13]. In line with this, Li et al. [18] also studied the significant effects of different environmental conditions on the leaf shape (leaf morphology) for the different varieties of *Z. jujuba*, which also suggests that *Ziziphus* species respond to changing environmental factors, i.e., nutrient provision and light intensity, in terms of their leaf morphology. Our results showed the morphological variation in the studied qualitative traits that could be utilized to optimize *Ziziphus* species in the Cholistan desert. Previously, Akter and Rehman [1] also characterized the morphological traits such as fruit shape, fruit apex, fruit base, and fruit color, leaf shape, leaf apex, leaf base, leaf margin, and leaf color for the determination of morphological differences in the different genotypes of *Z. mauritiana*. Moreover, Ivanišová et al. [45] performed an analysis regarding the morphological characteristics of the fruits and leaves for different genotypes of *Z. jujuba* grow in agroecological conditions in Ukraine.

In our results, the mean values of fruit length and fruit width of *Z. mauritiana* were noted as 2.53 cm and 2.55 cm, respectively. In similar research, the average fruit length and fruit width of *Z. mauritiana* were recorded as 1.60 cm and 1.16 cm by Yahia et al. [42]. They studied the features of *Z. mauritiana* under arid conditions. Variations in *Ziziphus* morphology could be attributed to different sampling sites and ecological effects. Similarly, the fruit size, leaf size, and petiole length of *Z. spina-christi* and *Z. nummularia* were larger than the previous findings under semi-arid conditions. This higher phenotypic diversity among the *Ziziphus* species can result from their cross and self-incompatibility characteristics [2]. Furthermore, Baghazadeh-Daryaiia et al. [46] also evaluated the petiole length, leaf size, and fruit pulp weight for *Z. spina-christi* in high rainfall conditions to understand the differences between the plant morphologies. In addition, Sabaghzadeh and Morid [47] also measured some values for the fruit diameter (23.9 mm) and fruit length (39.4 mm) for *Ziziphus mauritiana* under a cold semi-arid climate. These morphological differences were allied with changing climatic conditions, immigration, and proximity. Kumar et al. [48] found that fruit content depends on environmental and genetic factors, as the good quality seed is responsible for higher pulp weight and fruit size, which otherwise relies on the size of the seed. In *Ziziphus* plants, fruit with high weight is a supreme fruit character, while ecological conditions and cultivars can greatly influence the fruit weight. Our results were in accordance with the previous findings, which reported 78–83% moisture content in fruits of *Z. jujuba* cultivars [49]. Similarly, the difference in the fruit weight in *Ziziphus* plants was also reported by [50,51], who calculated the different weights of fruit and seed, which estimates high variability in *Ziziphus* plants. Variations in the fruit weight of *Z. jujuba* genotypes in different agroecological zones were reported previously by many scientists [52,53]. The difference in the weight of fruits from similar geographical areas may be a consequence of genotypic effects [54]. Prasad and Bankar [55] reported that genotypes with bigger and smaller sizes were responsible for varying the fruit weight in *Ziziphus*. In addition, the leaves of terrestrial plants are highly diverse and very sensitive to climate (high temperature and low rainfall). For instance, the variations in leaf traits, i.e., leaf size and leaf shape, are strongly correlated with the availability of light quantity and quality, and mean annual precipitation [18]. Therefore, we can conclude that (1) the extreme weather conditions, i.e., high temperature and low rainfall, significantly affect the morphological, physiological, and biochemical traits of *Ziziphus* species present in deserts; and (2) these types of extreme weather conditions favor the accumulation of some important biochemicals (flavonoids and phenols) in fruits and leaves of *Ziziphus* species, which are the primary source of medicine for local people living in these regions.

However, interestingly, the stressful conditions (desert) significantly improved the phytochemistry of *Ziziphus* species and increased the accumulation of phenols and flavonoids in leaves and fruits; it also enhanced the antioxidant activity (measured as DPPH) in all *Ziziphus* species, suggesting that *Ziziphus* species cope with water stress under desert conditions through increased antioxidant activity, which ultimately increases their medicinal and nutritional value. Results revealed that desert conditions significantly affect the total phenol and flavonoid content in *Ziziphus* plants. Our findings with phenolic content (fruit 214.20–309.23 mg GAE/100 g) and flavonoid content (fruits 98.06–127.41 mg QE/100 g and leaves 91.13–177.57 mg QE/100 g) were much higher than the previous results reported by Yahia et al. [42]; they determined the range of phenolic content in fruit as 148.75 to 293.46 mg GAE/100 g. In comparison, flavonoid content ranged from 21.21 to 46.51 mg QE/100 g and 90.26 to 92.22 mg QE/100 g in the fruits and leaves of two *Ziziphus* species, respectively. In *Ziziphus* leaves, flavonoid contents are located in the cuticula and epidermis, resulting in higher flavonoid content in leaves than fruits. Similarly, our results with flavonoid content in fruits ranged between 98.06 and 127.41 mg QE/100 g are greater than the previous findings of Cosmulescu et al. [41], where the total flavonoid content in full mature fruit was given as 26.27 and 19.9 mg QE/100 g in two cultivars of *Ziziphus jujuba*. Variations in the bioactive contents can be affected by many factors, i.e., fruit maturity, geographical locations, cultivar type, and other horticulture factors. *Ziziphus* species can grow well under dry conditions and high temperatures. Their fruit quality is best under sunny, dry, and hot conditions. In contrast, tropical plants with a higher content of flavonoids rely on the availability of light, while the amount of phenol depends mainly on different geographical conditions [42]. Furthermore, the amount of flavonoid content is related to the size of the *Ziziphus* fruits [46].

Similarly, Memon et al. [56] also reported the different values of the phenolic amount in ber fruit (*Z. mauritiana*), i.e., 12.8 mg GAE/1 g. Moreover, Ashraf et al. [57] also recorded the variation of flavonoid and phenol content in different leaf extracts of *Ziziphus* plants. They found the highest value for phenol in chloroform extract, while the maximum amount of flavonoid was noted in methanol extract. Differences in the quantity of flavonoids and phenols in various parts of *Ziziphus* species may be linked to nature, availability, and solubility of the chemical compound being extracted. Water and carbon fluctuations in the leaves can influence the morphological, physiological, developmental, and biochemical mechanisms of the plants, which are important for the preparation of primary and secondary metabolites, and also provide mechanical stability to the plants. Photosynthesis in the leaves of *Ziziphus* plants is very sensitive to water scarcity, which significantly affects the productivity and development of the plant [18]. Therefore, combined with the previous findings, our results also showed that the different *Ziziphus* species bear different phytochemical profiles as the phenols and flavonoid levels varied between all the studied species.

Antioxidant activity differences were also reported by Ashraf et al. [57], where the leaf extracts of *Ziziphus mauritiana* were analyzed to evaluate antioxidants. Different solvents (methanol, chloroform, and hexane) with different concentrations (mg/mL) were used to prepare leaf extracts. Methanol extract with 0.4 and 0.8 (mg/mL) concentrations revealed the highest percentage inhibition of DPPH. The leaf extract of *Ziziphus mauritiana* was observed to be endowed with the highest DPPH activities with an increase in concentration (mg/mL). At the same time, the percentage inhibition of DPPH can also be strongly influenced by the procedure of solvent extraction. Besides, variations in the antioxidants potential were also attributed to the different ripening stages of *Ziziphus* fruits measured by Cosmulescu et al. [41], where the antioxidant values ranged from 1661.4 to 1154.6 (mg acid ascorbic/100 g) in different cultivars of *Ziziphus jujuba*. Current findings agree well with the previous results reported by Yahia et al. [42], who indicated higher values of antioxidant potential in leaves than fruits of *Ziziphus lotus* and *Ziziphus mauritiana* due to the high number of bioactive compounds, i.e., polyphenols including flavonoids and tannins in the genus *Ziziphus*. Moreover, our findings follow the earlier studies, which endorse the high degree of variability between the *Ziziphus* species [58,59,60]. Such variation of antioxidants contents in different parts of *Ziziphus* plants mainly rely on the diverse weather conditions, i.e., temperature and rainfall [58].

## 5. Conclusions

Present inquiries outspread the different arrangements of morphological characters to recognize the distinctive morpho-types among the *Ziziphus* species. The results indicated the significant impact of different water conditions on the morphology of four *Ziziphus* species. Under irrigated conditions, *Ziziphus* species showed better morphology as compared to desert conditions. Observed results explored the high interspecific morphological differences among the species studied. *Ziziphus* includes highly marked and easily recognized plant individuals. Intraspecific diversity within species was also highly variant, which could be attributed to ecological effects and different growing conditions. We find that this study provides a vast range of variations in the phenols, flavonoids, and antioxidant activities in the fruits and leaves of the understudied *Ziziphus* species. Further, a higher amount of bioactive compounds in fruits and leaves of *Ziziphus* species under desert conditions than irrigated conditions greatly highlights the impact of the desert on the phytochemistry of the *Ziziphus* plants. However, further research is required to understand the resource capture mechanism of *Ziziphus* species in desert conditions, especially under the changing climate (high-temperature and low rainfall regions). A series of comprehensive studies are required to identify the physiological and biochemical mechanisms responsible for the accumulation of important phytochemicals in *Ziziphus* species.

## Figures and Tables

**Table 1:** Qualitative morphological characters of fruits of *Ziziphus* species.

	Desert				Irrigated			
Parameters	*Z. jujuba*	*Z. mauritiana*	*Z. spina*-*christi*	*Z. nummularia*	*Z. jujuba*	*Z. mauritiana*	*Z. spina-christi*	*Z. nummularia*
Shape	Oval	Orbicular	Oval	Orbicular	Oval, rounded	Rounded, ovate	Oval, rounded	Orbicular
Apex	Retuse	Oblate	Obtuse	Oblate	Retuse, truncate	Rounded, oblate	Obtuse	Oblate
Base	Rounded	Flattened	Rounded	Flattened	Rounded, oval	Orbicular	Rounded	Flattened, rounded
Color	Yellowish green to red	Dark green	Light green to yellow	Red, yellow, green	Yellowish green to red	Dark green	Light green to yellow	Red, yellow, green
Stalk color	Light green	Green	Light green	Light green	Light green	Light green, green	Light green	Light green, green

**Table 2:** Qualitative morphological characters of leaves of Ziziphus species.

	Desert				Irrigated			
Parameters	*Z. jujuba*	*Z. mauritiana*	*Z. spina-christi*	*Z. nummularia*	*Z. jujuba*	*Z. mauritiana*	*Z. spina-christi*	*Z. nummularia*
Shape	Oval, oblong	Oval, lanceolate	Oval, ovate, cordate	Oval, rounded	Oval, oblong	Oval, ovate, cordate	Oval, ovate	Rounded, ovate
Apex	Obtuse	Obtuse	Obtuse, acute	Obtuse	Obtuse	Obtuse	Obtuse, acute	Obtuse
Base	Rounded, acute	Rounded, acute	Cordate, rounded	Rounded	Cuneate, rounded	Acute, rounded	Cordate, rounded	Rounded
Margin	Entire	Entire	Entire	Entire	Entire	Entire	Entire	Entire
Veins	Prominent	Branched	Branched	Less prominent	Prominent	Much branched	Less branched	Less prominent
Color	Shining dark green	Light green	Dull green	Less dull green	light and dark shining green	Light and dark green	Dull green	Less dull shining green
Petiole surface	Pubescent	Hairs	Hairs	Glabrous	Pubescent	Hairs	Hairs	Glabrous
Petiole color	Green	Red, green	Green, red and yellow	Red	Green	Red, green	Green, red and yellow	Red

**Table 3:** Mean value of some quantitative characters for fruits of *Ziziphus* species.

		Fruit Length (cm)	Fruit Width (cm)	Fruit Diameter (cm)	Fruit Area (cm^3^)	Fruit Stalk Length (cm)	Fruit dry Weight (%)	Seed Length (cm)	Seed Width (cm)	Seed Diameter (cm)
**Conditions (C)**	**Irrigated**	2.7 a	2.8 a	2.8 a	38.1 a	1.2 a	32.2 a	1.6 a	1.4 a	1.0 a
	**Desert**	2.5 b	2.6 b	2.6 b	36.1 b	1.2 a	26.5 b	1.4 b	1.2 b	0.9 b
**LSD (0.05)**		7.2	6.1	0.02	1.6	0.02	1.3	0.03	0.03	0.02
**Species (S)**	**S1**	3.9 a	3.8 a	3.6 a	66.1 a	1.5 a	14.9 d	2.0 a	1.3 b	1.1 a
	**S2**	2.6 b	2.5 b	2.5 c	31.0 c	1.0 c	29. 1 b	1.5 b	1.3 b	1.1 a
	**S3**	2.5 c	2.3 c	2.7 b	33.1 b	1.2 b	24.4 c	1.3 c	1.3 b	0.8 b
	**S4**	1.5 d	2.0 d	2.0 d	17.8 d	1.0 c	48.9 a	1.0 d	1.4 a	0.8 b
**LSD (0.05)**		0.03	0.02	0.03	1.9	0.01	0.9	0.02	0.01	0.2
**Interaction**	**(C × S)**	*	*	NS	NS	*	NS	NS	*	*

S1, S2, S3, and S4 represent four *Ziziphus* species, i.e., *Z. jujuba*, *Z. mauritiana*, *Z. spina-christi*, and *Z. nummularia*, respectively. Means are averaged over three replicates. Means that do not share the same letters in the column differ significantly at *p* ≤ 0.05. While the symbol * represents significant interaction.

**Table 4:** Mean value of some quantitative characters for leaves of *Ziziphus* species.

.		Leaf Length (cm)	Leaf Width (cm)	Leaf Area (cm^3^)	Leaf Petiole Length (cm)	Leaf Dry Weight (%)
**Conditions (C)**	Irrigated	4.9 a	3.2 a	17.9 a	1.0 a	50. 5 a
	Desert	4.8 a	3.1 b	16.3 b	0.9 b	43.9 b
LSD (0.05)		0.4	0.07	0.8	0.03	0.9
Species (S)	S1	7.4 a	4.2 a	30.6 a	1.3 a	45.6 c
	S2	5.1 b	3.0 c	16.5 b	1.3 a	55. 4 a
	S3	4.5 c	3.4 b	15.8 b	0.8 b	37.7 d
	S4	2.6 d	2.1 d	5.5 c	0.5 c	50.1 b
LSD (0.05)		0.2	0.1	0.9	0.04	1.2
Interaction	(C × S)	NS	NS	NS	NS	NS

S1, S2, S3, and S4 represent four *Ziziphus* species, i.e., *Z. jujuba*, *Z. mauritiana*, *Z. spina-christi*, and *Z. nummularia*, respectively. Means are averaged over three replicates. Means that do not share the same letters in the column differ significantly at *p* ≤ 0.05.

**Table 5:** Total phenol contents, total flavonoid contents, and % inhibition of DPPH scavenger activities in the fruits and leaves of *Ziziphus* species.

		Total Phenol Fruit (mg GAE/100 g)	Total Phenol Leaves (mg GAE/100 g)	Total Flavonoids Fruit (mg QE/100 g)	Total Flavonoids Leaves (mg QE/100 g)	Fruits (% Inhibition of DPPH)	Leaves (% Inhibition of DPPH)
Conditions (C)	Irrigated	261.8 b	271.1 b	102.4 b	98.0 b	53.2 b	58.5 b
	Desert	272.0 a	280.9 a	108.0 a	104.1 a	65.4 a	69.0 a
LSD (0.05)		0.3	0.9	1.2	1.4	11.0	3.6
Species (S)	S1	284.6 b	296.9 b	98.7 c	94.3 c	59.6 b	64.8 b
	S2	207.6 d	234.4 d	102.9 b	108.9 b	57.7 c	62.5 c
	S3	271.0 c	258.4 c	95.6 d	87.9 d	66.7 a	70.7 a
	S4	304.4 a	314.2 a	123.7 a	113.4 a	53.1 d	57.1 d
LSD (0.05)		1.4	1.2	1.9	2.2	1.4	1.7
Interaction	(C × S)	*	*	NS	NS	*	NS

S1, S2, S3, and S4 represent four *Ziziphus* species, i.e., *Z. jujuba*, *Z. mauritiana*, *Z. spina-christi*, and *Z. nummularia*, respectively. Means are averaged over three replicates. Means that do not share the same letters in the column differ significantly at *p* ≤ 0.05. While the symbol * represents significant interaction.

## Data Availability

Not applicable.

## References

[1] Akter A., Rahman H. (2019). Characterization of Ber (*Ziziphus mauritiana*) Genotypes. J. Agric. Sci. Technol..

[2] Norouzi E., Erfani-Moghadam J., Fazeli A., Khadivi A. (2017). Morphological variability within and among three species of *Ziziphus* genus using multivariate analysis. Sci. Hortic..

[3] Islam M.B., Guralnick R.P. (2015). Generic placement of the former *Condaliopsis* (Rhamnaceae) species. Phytotaxa.

[4] Hauenschild F., Matuszak S., Muellner-Riehl A.N., Favre A. (2016). Phylogenetic relationships within the cosmopolitan buckthorn family (Rhamnaceae) support the resurrection of *Sarcomphalus* and the description of *Pseudoziziphus* gen. nov. Taxon.

[5] Suliman M.B., Mohammed A.A. (2018). Preliminary phytochemical screening and antibacterial activity of ethanolic and aqueous extracts of Sudanese medicinal plant *Ziziphus spina-christi* L leaves. Arab. J. Med. Aromat. Plants.

[6] Nyanga L.K., Gadaga T.H., Nout M.J., Smid E.J., Boekhout T., Zwietering M.H. (2013). Nutritive value of masau (*Ziziphus mauritiana*) fruits from Zambezi Valley in Zimbabwe. Food Chem..

[7] Orwa C. (2009). Agroforestree Database: A Tree Reference and Selection Guide, Version 4.0. http://www.worldagroforestry.org/sites/treedbs/treedatabases.

[8] Razi M., Anwar R., Basra S., Khan M.M., Khan I.A. (2013). Morphological characterization of leaves and fruit of jujube (*Ziziphus mauritiana* Lamk.) germplasm in Faisalabad, Pakistan. Pak. J. Agric. Sci..

[9] Alhassan K.A., Indabawa A.S., Shah M.M. (2019). Phytochemical analysis, proximate composition and antibacterial activities of *Ziziphus* species (*Z. jujube* and *Z. spina-christi*). J. Appl. Adv. Res..

[10] Beg M.A., Teotia U., Farooq S. (2016). In vitro antibacterial and anticancer activity of Ziziphus. J. Med. Plants Stud..

[11] Arbonnier M. (2004). Trees, Shrubs and Lianas of West African Dry Zones.

[12] El Amin H.M. (1990). Trees and Shrubs of the Sudan.

[13] Saied A.S., Gebauer J., Hammer K., Buerkert A. (2008). Ziziphus spina-christi (L.) Willd.: A multipurpose fruit tree. Genet. Resour. Crop Evol..

[14] Diaz S., Cabido M., Casanoves F. (1998). Plant functional traits and environmental filters at a regional scale. J. Veg. Sci..

[15] Garnier J., Billen G. (2002). The Riverstrahler modelling approach applied to a tropical case study (The Red-Hong-River, Vietnam): Nutrient transfer and impact on the Coastal Zone. Coll. Mar. Res. Works.

[16] Walter A., Schurr U. (2005). Dynamics of leaf and root growth: Endogenous control versus environmental impact. Ann. Bot..

[17] Paroda R., Mal B., Wickens G.E., Haq N., Day P. (1989). New plant sources for food and industry in India. New Crops for Food and Industry.

[18] Li X., Li Y., Zhang Z., Li X. (2015). Influences of environmental factors on leaf morphology of Chinese jujubes. PLoS ONE.

[19] Wolfe A.D., Liston A. (1998). Contributions of PCR-based methods to plant systematics and evolutionary biology. Molecular Systematics of Plants II.

[20] Royer D.L., McElwain J.C., Adams J.M., Wilf P. (2008). Sensitivity of leaf size and shape to climate within *Acer rubrum* and *Quercus kelloggii*. New Phytol..

[21] Jones C.S. (1995). Does shade prolong juvenile development? A morphological analysis of leaf shape changes in *Cucurbita argyrosperma* Subsp. *Sororia* (Cucurbitaceae). Am. J. Bot..

[22] Du N., Wang R., Liu J., Zhang X., Tan X., Wang W., Chen H., Guo W. (2013). Morphological response of *Vitex negundo* var. *heterophylla* and *Ziziphus jujuba* var. *spinosa* to the combined impact of drought and shade. Agrofor. Syst..

[23] Goswami P., Banerjee R., Mukherjee A. (2019). Potential antigenotoxicity assessment of *Ziziphus jujuba* fruit. Heliyon.

[24] Masullo M., Montoro P., Autore G., Marzocco S., Pizza C., Piacente S. (2015). Quali-quantitative determination of triterpenic acids of *Ziziphus jujuba* fruits and evaluation of their capability to interfere in macrophages activation inhibiting NO release and iNOS expression. Food Res. Int..

[25] Song L., Liu P., Yan Y., Huang Y., Bai B., Hou X., Zhang L. (2019). Supercritical CO_2_ fluid extraction of flavonoid compounds from Xinjiang jujube (*Ziziphus jujuba* Mill.) leaves and associated biological activities and flavonoid compositions. Ind. Crops Prod..

[26] Bai L., Zhang H., Liu Q., Zhao Y., Cui X., Guo S., Zhang L., Ho C.-T., Bai N. (2016). Chemical characterization of the main bioactive constituents from fruits of *Ziziphus jujuba*. Food Funct..

[27] Ahmad M., Zafar M., Sultana S. (2009). *Salvadora persica*, *Tamarix aphylla* and *Zizyphus mauritiana*-Three woody plant species mentioned in Holy Quran and Ahadith and their ethnobotanical uses in north western part (DI Khan) of Pakistan. Pak. J. Nutr..

[28] Hussain S.M., Khan A., Khan A.-u., Qazi N.G., Ali F. (2017). Pharmacological basis for medicinal use of *Ziziphyus nummularia* (Rhamnaceae) leaves in gastrointestinal disorders. Trop. J. Pharm. Res..

[29] Zhang R., Chen J., Shi Q., Li Z., Peng Z., Zheng L., Wang X. (2014). Phytochemical analysis of Chinese commercial *Ziziphus jujube* leaf tea using high performance liquid chromatography–electrospray ionization-time of flight mass spectrometry. Food Res. Int..

[30] Li D., Yue D., Liu D., Zhang L., Song S. (2020). Phytochemical and chemotaxonomic study on *Ziziphus jujuba* Mill.(Rhamnaceae). Biochem. Syst. Ecol..

[31] Dalleau S., Baradat M., Guéraud F., Huc L. (2013). Cell death and diseases related to oxidative stress: 4-hydroxynonenal (HNE) in the balance. Cell Death Differ..

[32] Lobo V., Patil A., Phatak A., Chandra N. (2010). Free radicals, antioxidants and functional foods: Impact on human health. Pharmacogn. Rev..

[33] Khadri A., Neffati M., Smiti S., Falé P., Lino A.R.L., Serralheiro M.L.M., Araújo M.E.M. (2010). Antioxidant, antiacetylcholinesterase and antimicrobial activities of *Cymbopogon schoenanthus* L. Spreng (lemon grass) from Tunisia. LWT-Food Sci. Technol..

[34] Duthie G., Morrice P. (2012). Antioxidant capacity of flavonoids in hepatic microsomes is not reflected by antioxidant effects in vivo. Oxidative Med. Cell. Longev..

[35] Huang W.-Y., Cai Y.-Z., Zhang Y. (2009). Natural phenolic compounds from medicinal herbs and dietary plants: Potential use for cancer prevention. Nutr. Cancer.

[36] Pandey K.B., Rizvi S.I. (2009). Plant polyphenols as dietary antioxidants in human health and disease. Oxidative Med. Cell. Longev..

[37] Farooq Z., Iqbal Z., Mushtaq S., Muhammad G., Iqbal M.Z., Arshad M. (2008). Ethnoveterinary practices for the treatment of parasitic diseases in livestock in Cholistan desert (Pakistan). J. Ethnopharmacol..

[38] Ahmed M.J., Murtaza G. (2015). A study of medicinal plants used as ethnoveterinary: Harnessing potential phytotherapy in Bheri, district Muzaffarabad (Pakistan). J. Ethnopharmacol..

[39] Hernández F., Noguera-Artiaga L., Burló F., Wojdyło A., Carbonell-Barrachina Á.A., Legua P. (2016). Physico-chemical, nutritional, and volatile composition and sensory profile of Spanish jujube (*Ziziphus jujuba* Mill.) fruits. J. Sci. Food Agric..

[40] Hussain T., Fatima I., Rafay M., Shabir S., Akram M., Bano S. (2016). Evaluation of antibacterial and antioxidant activity of leaves, fruit and bark of Kigelia africana. Pak. J. Bot..

[41] Cosmulescu S., Trandafir I., Violeta N., Achim G., Mihai B., Iordanescu O. (2018). Variation of bioactive compounds and antioxidant activity of jujube (*Ziziphus jujuba*) fruits at different stages of ripening. Not. Bot. Horti Agrobot. Cluj-Napoca.

[42] Yahia Y., Benabderrahim M.A., Tlili N., Bagues M., Nagaz K. (2020). Bioactive compounds, antioxidant and antimicrobial activities of extracts from different plant parts of two *Ziziphus* Mill. species. PLoS ONE.

[43] Zeinelabdin M. (2016). Variation of some morphological characteristics of *Ziziphus spina-chriti* (A.) Ritch. fruits among four provenances. Int. J. Sci. Res. Publ..

[44] Almalki R.A., Alzahrani D.A. (2018). Morphological Investigation of Genus *Ziziphus* Mill.(Rhamnaceae) in Saudi Arabia. Am. J. Plant Sci..

[45] Ivanišová E., Grygorieva O., Abrahamova V., Schubertova Z., Terentjeva M., Brindza J. (2017). Characterization of morphological parameters and biological activity of jujube fruit (*Ziziphus jujuba* Mill.). J. Berry Res..

[46] Baghazadeh-Daryaii L., Sharifi-Sirchi G.-R., Samsampoor D. (2017). Morphological, phytochemical and genetic diversity of *Ziziphus spina–christi* (L) Des. in South and Southeastern of Iran. J. Appl. Res. Med. Aromat. Plants.

[47] Sabaghzadeh F., Morid A. (2014). Comparison above genotypes of *Ziziphus* species (*Ziziphus spina-christi* & *Ziziphus mauritania*) extant in Dezfoul fadak botanical garden used fruit and seed traits. Agric. Sci. J..

[48] Kumar V., Ajeesh R., Vidyasagaran K., Babu S. (2015). Variation in pulp content and physical characters of *Elaeocarpus serratus* L. Fruits in different landuse patterens of Western Ghats, India. Ecol. Environ. Conserv..

[49] Gao Q.-H., Wu P.-T., Liu J.-R., Wu C.-S., Parry J.W., Wang M. (2011). Physico-chemical properties and antioxidant capacity of different jujube (*Ziziphus jujuba* Mill.) cultivars grown in loess plateau of China. Sci. Hortic..

[50] Markovski A., Velkoska-Markovska L. (2015). Investigation of the morphometric characteristics of jujube types (*Zizyphus jujuba* Mill.) fruits in Republic of Macedonia. Genetika.

[51] Azam-Ali S., Bonkoungou E., Bowe C., DeKock C., Godara A., Williams J. (2001). Fruits for the Future-2 (Revised Edition), Ber and Other Jujubes.

[52] Karnatovska M., Brindza J., Grygorieva O., Derevjanko V., Kochanova Z., Birova D. (2007). Jujube Fruit (*Zizyphus jujuba* Mill.) Variability Determination. Proceedings of the 1st International Scientific Conference on Medicinal, Aromatic and Spice Plants Book of Scientific Papers and Abstracts.

[53] Brindza J., Karnatovska M., Grygorieva O., Vietoris V., Kucelová L., Erdélyová G. (2011). Morphological and organoleptic nature of *Ziziphus jujuba* Mill. Potravin. Slovak J. Food Sci..

[54] Karadeniz T. (2002). Selection of native ‘Cornelian’ cherries grown in Turkey. J. Am. Pomol. Soc..

[55] Prasad R., Bankar G. (2000). Evaluation of pomegranate cultivars under arid conditions. Indian J. Hortic..

[56] Memon A.A., Memon N., Luthria D.L., Pitafi A.A., Bhanger M.I. (2012). Phenolic compounds and seed oil composition of *Ziziphus mauritiana* L. fruit. Pol. J. Food Nutr. Sci..

[57] Ashraf A., Sarfraz R.A., Anwar F., Shahid S.A., Alkharfy K.M. (2015). Chemical composition and biological activities of leaves of *Ziziphus mauritiana* L. native to Pakistan. Pak. J. Bot..

[58] Hossain M.A. (2019). A phytopharmacological review on the Omani medicinal plant: *Ziziphus jujube*. J. King Saud Univ. Sci..

[59] Esteki T., Urooj A. (2012). Antioxidant components and activity in the peel of *Ziziphus Jujuba* mill. J. Pharm. Res..

[60] Al-Saeedi A.H., Al-Ghafri M.T.H., Hossain M.A. (2016). Comparative evaluation of total phenols, flavonoids content and antioxidant potential of leaf and fruit extracts of Omani *Ziziphus jujuba* L.. Pac. Sci. Rev. Nat. Sci. Eng..

